# Antimicrobial Activity of Fermented Vegetable Byproduct Extracts for Food Applications

**DOI:** 10.3390/foods10051092

**Published:** 2021-05-14

**Authors:** Annalisa Ricci, Gaia Bertani, Antonietta Maoloni, Valentina Bernini, Alessia Levante, Erasmo Neviani, Camilla Lazzi

**Affiliations:** 1 Department of Food and Drug, University of Parma, Parco Area delle Scienze 49/A, 43124 Parma, Italy; annalisa.ricci@unipr.it (A.R.); gaia.bertani@unipr.it (G.B.); alessia.levante@unipr.it (A.L.); erasmo.neviani@unipr.it (E.N.); camilla.lazzi@unipr.it (C.L.); 2 Dipartimento di Scienze Agrarie, Alimentari ed Ambientali, Università Politecnica delle Marche, Via Brecce Bianche, 60131 Ancona, Italy; a.maoloni@pm.univpm.it; 3 SITEIA.PARMA—Centro Interdipartimentale sulla Sicurezza, Tecnologie e Innovazione Agroalimentare, University of Parma, Tecnopolo Pad. 33 Campus Universitario, 43124 Parma, Italy

**Keywords:** fermented byproduct extracts, minimum bactericidal concentration, challenge test, minced meat, foodborne pathogens

## Abstract

To prevent foodborne diseases and extend shelf-life, antimicrobial agents may be used in food to inhibit the growth of undesired microorganisms. In addition to the prevention of foodborne diseases, another huge concern of our time is the recovery of agri-food byproducts. In compliance with these challenges, the aim of this work was to more deeply investigate the antimicrobial activity of extracts derived from fermented tomato, melon, and carrot byproducts, previously studied. All the fermented extracts had antimicrobial activity both in vitro and in foodstuff, showing even higher activity than commercial preservatives, tested for comparison against spoilage microorganisms and foodborne pathogens such as *Salmonella* spp., *L. monocytogenes*, and *B. cereus*. These promising results highlight an unstudied aspect for the production of innovative natural preservatives, exploitable to improve the safety and shelf-life of various categories of foodstuff.

## 1. Introduction

Foodborne diseases have been an important health problem for all populations since the beginning of humanity. The types, severity, and impacts of these illnesses have changed over time and widely differ across regions, countries, and communities. All over the world, foodborne diseases are an important cause of morbidity and mortality, as well as an important impediment to socioeconomic development. Billions of people are at risk, and millions fall ill every year as the result of consuming unsafe food [1]. Among the microorganisms most relevant and most frequently notified by the RASFF in the last 40 years can be found *Salmonella* spp., *Listeria monocytogenes*, *Escherichia coli*, *Staphylococcus aureus*, and *Bacillus cereus* [2,3]. Moreover, nonpathogenic microorganisms, such as *Pseudomonas* spp., can be considered a serious problem because they can reduce the quality of contaminated food products [4]. To avoid foodborne disease risk and food deterioration, different methods for preserving food have been applied until today. Among them, antimicrobials may be used to upgrade food product safety by inhibiting/inactivating pathogenic microorganisms or by improving the shelf-life of food perishable by spoilage microorganisms [5]. Currently, consumers have concerns about the synthetic preservatives used in food [6,7]. Accordingly, an increasing number of consumers are demanding minimally processed foods and “cleaner” labels, leading to a great interest in natural antimicrobial utilization [5]. In the same direction, in recent years, there has been a growing increase in research concerning the investigation of antimicrobials from different natural sources. In fact, natural antimicrobials can be obtained from different sources such as plants [8,9,10], animals, bacteria, algae, and fungi [11,12]; however, considering the wide biodiversity of this biological heritage, there is still much research to be done [6,13]. An important source of natural antimicrobials can be represented by plant-derived compounds or extracts. In addition, byproduct extracts have been tested, and their efficacy was documented in Gram-positive and Gram-negative pathogens, as well as yeast, fungi, and mold. Extracts obtained from different fruit and vegetable byproducts such as peels, husks, seeds, or leaves were tested against various foodborne pathogens such as *Salmonella* spp., *L. monocytogenes*, *E. coli*, *S. aureus*, and *B. cereus* and spoilage microorganisms such as *Pseudomonas* spp. [14].

Fermentation can also be a valid and successful technology for the production of compounds inhibiting foodborne microorganism growth [15,16,17]. It is a traditional method for food processing that extends shelf-life and, simultaneously, improves organoleptic properties. In food productions, lactic acid fermentation is a technique largely used as lactic acid bacteria (LAB) are generally recognized as safe (GRAS) microorganisms able to produce antimicrobial compounds such as organic acids (lactic, acetic, etc.), diacetyl, bacteriocins, and other metabolites [15]. At the same time, vegetable and fruit processing generates a large number of byproducts still rich in nutrients and bioactive compounds, which may be fermented and metabolized by microorganisms. The ability of some microorganisms to use substrates deriving from vegetable and food waste can be advantageous. Indeed, on a global scale, one-third of food produced (corresponding to about 1.3 billion metric tons of food per year) is lost or wasted [18] and, in the European Union alone, 90 million metric tons of food waste is generated annually [19]. Byproducts can be classified into different groups on the basis of the steps of the agri-food chain in which they are generated: in field, before harvesting, postharvest, during transport, during manufacturing, on the market, and in the consumer’s house. Considering these steps during the life of a foodstuff, byproducts can be represented by damaged crops, fruits or vegetables with inadequate size or ripening, and peels, seeds, or residues deriving from the industrial processing or discarded during retail or by consumers. However, the employment of byproducts can also present some issues such as perishability, due to the high content of nutrients and water, seasonality, limited availability of a specific by-product, or distribution across different territories, with a consequent heterogeneity of the products, which can hinder the employment of agri-food byproducts [20].

Therefore, in the context of the circular economy, fermentation can be a valid strategy to exploit agri-food byproducts as a potential source of low-cost substrates for fermentation and for natural antimicrobial recovery [15]. In the present work, the antimicrobial activity of fermented tomato, melon, and carrot byproduct extracts, already reported in a previous study [15], was more deeply investigated. The minimum bactericidal concentration (MBC) of extracts was determined for 16 strains belonging to *Salmonella* spp., *L. monocytogenes*, *E. coli*, *S. aureus*, *B. cereus*, and *Pseudomonas* spp. Moreover, the antimicrobial activity against spoilage microorganisms exerted by fermented byproduct extracts was tested using minced meat and ready-to-eat sliced vegetal products as food models. Lastly, microbiological challenge tests were performed to monitor the behavior of foodborne pathogens in the presence of fermented byproduct extracts during storage.

## 2. Materials and Methods

### 2.1. Byproduct Fermentation and Extract Production

Tomato, melon, and carrot byproducts were recovered by local farmers located in the Emilia Romagna Region (Italy), ground using an Oster 809-48H mixer (Recampro, Rianxo, Spain) until they became a mush, sterilized at 121 °C for 20 min, and stored in glass jars in an ultra-low-temperature (−80 °C) freezer (U725, Innova, New Brunswick, Eppendorf, Hamburg, Germany) for a maximum of 4 months. Tomato byproducts were composed of peels and seeds obtained after the industrial processing of tomato fruits, whereas melon and carrot byproducts were those products unsuitable for retail because of their ripeness or size or because they were damaged. *Lacticaseibacillus rhamnosus* 1473, *Lacticaseibacillus casei* 2240, and *Lacticaseibacillus casei* 2246, belonging to the collection of the Food and Drug Department (University of Parma, Parma, Italy), were used as starters for tomato, melon, and carrot byproduct fermentation, respectively, as reported by Ricci et al. (2019) [15]. Briefly, the three sterilized substrates (tomato, melon, and carrot byproducts) were inoculated individually with the mentioned strain suspensions in order to obtain a final concentration of 7 log_10_ CFU/mL. The inoculated byproducts were then incubated for 72 h at 37 °C. Fermented byproducts were then lyophilized and subjected to water/ethanol 50/50 (*v*/*v*) extraction as previously reported [15]. The extracts obtained were freeze-dried in order to obtain a powder and stored at −80 °C until their use.

### 2.2. Microorganisms Tested

The antimicrobial activity of extracts was tested toward 16 strains belonging to *Salmonella* spp. (*S. enterica* ATCC 14028, *S. enterica* serotype Rissen, *Salmonella* spp. suini), *L. monocytogenes* (LM30, LMG 21264, LMG 13305), *E. coli* (DSMZ 9025, DSMZ 10973, POM 1048), *S. aureus* (NCTC 9393, ATCC 6538, ATCC 19095), *B. cereus* (31), and *Pseudomonas* spp. (5003, 5004, 5005). The strains used came from the following collections: Deustsche Sammlung von Mikroorganismen und Zellkulturen GmbH (DSMZ), the American Type Culture Collection (ATCC), the Belgian Coordinated Collection of Microorganisms (BCCM/LMG), the National Collection of Type Cultures (NCTC), and the collection of the Food and Drug Department (University of Parma, Parma, Italy). All strains were kept at −80 °C in tryptic soy broth (TSB) (Oxoid, Milan, Italy) supplemented with 12.5% glycerol (*v*/*v*). Before use, they were cultured twice for 24 h at 37 °C (*Salmonella* spp., *L. monocytogenes*, *E. coli*, *S. aureus*, and *B. cereus*) and 30 °C (*Pseudomonas* spp.) with a 3% *v*/*v* inoculum in TSB added with 0.6% yeast extract (Oxoid, Milan, Italy). Each revitalized culture was used to test the susceptibility to the fermented byproduct extracts.

### 2.3. Susceptibility Tests

The minimum inhibitory concentration (MIC) of fermented byproduct extracts was determined using the microdilution method according to Ricci et al. (2016) [21] with some modifications. Briefly, TSB was used, and microplates were incubated for 24 h at the optimal growth temperature tested. Due to the turbidity of extracts, the MIC determination was prone to error; therefore, all the dilutions were re-cultured (10 µL) on Tryptic Soy Agar (TSA) (Oxoid, Milan, Italy) and incubated at the same temperature for 24 h to determine the MBC [22]. Different microbial concentrations were tested (approximately 8 log_10_ CFU/mL, 6 log_10_ CFU/mL, 4 log_10_ CFU/mL, and 2 log_10_ CFU/mL).

### 2.4. Effect of Fermented Byproduct Extracts on Food Preservation

The antimicrobial activity against spoilage microorganisms exerted by fermented byproduct extracts was tested using minced meat and ready-to-eat sliced vegetal products as food models. These food models were selected according to their origin, both animal and vegetal, the high perishability that could make the use of an antimicrobial attractive, and the simplicity of the formulation that allowed a homogeneous distribution of the extract on a laboratory scale. Minced pork meat was bought from a local market and used to test the antimicrobial activity of fermented tomato byproduct extract. Different concentrations of extract were tested (0.8% (*w*/*w*), 1.2% (*w*/*w*), 1.6% (*w*/*w*), and 2.4% (*w*/*w*)) and, at the same time, compared with the most applied preservatives in meat (sodium lactate 1.2% (*w*/*w*) and sodium lactate/sodium diacetate (96:4) 2.5% (*w*/*w*)). The concentrations tested “in situ” were chosen in a range near the concentrations of common preservatives tested, lower than the MBC. The extract/preservative was added to the minced meat and mixed. Small hamburgers of 10 g were prepared and stored at refrigerated temperature. Total microbial count was analyzed immediately after the addition of extract/preservative (T0) and after 3 (T3), 6 (T6), and 9 (T9) days of storage. On the other hand, a ready-to-eat sliced vegetal product, mainly composed of water, melon, and thickeners, produced by a local food industry (Salumificio San Paolo S.r.l, Traversetolo, PR, Italy), was used to test the antimicrobial activity of fermented melon and carrot byproduct extracts. The extracts were added separately at 1.2% (*w*/*w*) during the preparation of the vegetal product. Afterward, it was sliced and stored under vacuum in refrigerated conditions. Total bacterial load was detected after product preparation and solidification (T0) and after 15 (T15) and 30 (T30) days of storage. For both products, the analyses were conducted by homogenizing the samples with ringer solution (VWR, Milan, Italy); then, decimal dilutions were spread on plate count agar (PCA) (VWR, Milan, Italy) and incubated for 72 h at 30 °C (UNI EN ISO 4833-1:2013). In both experiments, the same process was followed without adding extracts, and these samples were used as control. All experiments were performed in triplicate.

### 2.5. Effect of Fermented Tomato Byproduct Extracts on Foodborne Pathogens in Minced Meat

Minced pork meat was also used as a food model to simulate, through microbiological challenge test, the behavior of different foodborne pathogens (*Salmonella* spp., *L. monocytogenes,* and *B. cereus)* in case of eventual contamination. Before artificial contamination of the samples, revitalized strains belonging to the same species were mixed in equal concentration. Minced pork meat was added with fermented tomato byproduct extract 1.6%, sodium lactate 1.2%, or sodium lactate/sodium diacetate (96:4) 2.5%, while one sample was used as a control without the addition of extract/preservative. The contamination was carried out under sterile conditions with high microbial concentrations, approximatively ranging between 5 and 6 log_10_ CFU/g in the product. All samples were stored in refrigerated conditions for 9 days, and the microbial contamination was checked just after the preparation of samples (T0) and after 3 (T3), 6 (T6), and 9 (T9) days. The experiments were performed in triplicate. Quantification of *Salmonella* spp., *L. monocytogenes*, and *B. cereus* was carried out at the scheduled time. The concentration of *Salmonella* spp. was detected using the medium Rambach agar (Merck, Milan, Italy), incubating the plates at 37 °C for 24 h. The determination of *L. monocytogenes* was performed by applying the indication reported in ISO 11290–2:2017 using *Listeria* selective agar base according to Ottaviani and Agosti (VWR, Milan, Italy). Enumeration of *B. cereus* was determined following the ISO 7932:2005 using a mannitol egg yolk polymyxin agar base (MYP) (VWR, Milan, Italy).

### 2.6. Statistical Analysis

One-way ANOVA was used to evaluate differences among treatments (addition or not of fermented byproduct extracts or preservatives) at the same storage time. The analyses were conducted with SPSS, version 21.0 software (IBM Corp., Armonk, NY, USA) using Tukey’s test; a *p*-value < 0.05 was considered statistically significant.

## 3. Results

### 3.1. Minimum Bactericidal Concentration

The MBC of fermented tomato, melon, and carrot byproduct extracts, which is the lowest concentration of extract responsible for the death of inoculated cells, was evaluated by considering four concentrations of the target microorganisms (Table 1). Regarding tomato byproduct extract, it had the highest activity against *E. coli, S. aureus, B. cereus*, and *Pseudomonas* spp. Overall, the tested strains had an MBC value of 25 mg/mL which decreased to 12.5 mg/mL at the lowest concentration of pathogen tested (2 log_10_ CFU/mL). *Salmonella* spp. showed the same susceptibility with the exception of the suini strain, which was less affected than the other strains analyzed. *L. monocytogenes* seemed to be the species least inhibited, especially at high concentration, while, at 2 log_10_ CFU/mL, the MBC was comparable with the other species studied. Melon byproduct extract, compared to the other extracts, revealed a more homogenous activity. Practically all the tested strains considered at diverse concentrations had the same MBC value (25 mg/mL) with the exception of two strains of *Pseudomonas* spp. (5003 and 5004), having the highest MBC at the highest concentration. The carrot byproduct fermented extract was the most effective (6.25 and 3.125 mg/mL) against all pathogens tested at the concentrations of 6, 4, and 2 log_10_ CFU/mL.

### 3.2. Effect of Fermented Byproduct Extracts on Food Preservation

**Figure 1:**
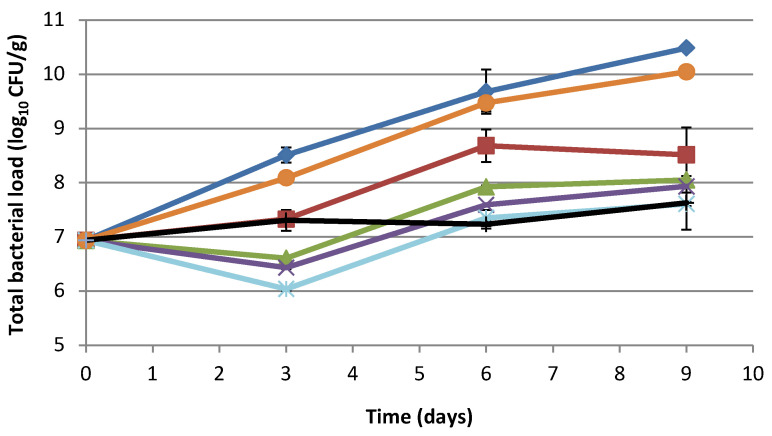
Effect of fermented byproduct extracts on food preservation. Total bacterial load (log_10_ CFU/g) in minced pork meat with or without fermented tomato byproduct extract/preservative. Minced meat (blue line) and minced meat with 0.8% fermented tomato byproduct extract (red line), 1.2% fermented tomato byproduct extract (green line), 1.6% fermented tomato byproduct extract (purple line), 2.4% fermented tomato byproduct extract (light blue line), 1.2% sodium lactate (orange line), and 2.5% sodium lactate/sodium diacetate (96:4) (black line).

Considering a possible application in food, we decided to evaluate the effect of fermented tomato byproduct extract in minced pork meat and of fermented melon and carrot byproduct extracts in a ready-to-eat sliced vegetal product. Total bacterial load was evaluated in minced pork meat with the presence or absence of tomato byproduct fermented extract, at different concentrations, or with diverse preservatives. The changes in microbial concentration were evaluated over 9 days in refrigerated storage (Figure 1). After 3 days (T_3_), the highest decrease in microbial concentration was observed in minced meat with 1.2%, 1.6%, and 2.4% tomato by-product extract compared to T_0_. In minced meat, untouched or with sodium lactate, an increase in total bacterial load of 1.57 log_10_ CFU/g and 1.15 log_10_ CFU/g was observed, respectively. On the other hand, the adjunction of 0.8% extract and sodium lactate/sodium diacetate showed little to no changes in the initial total microbial count (Figure 1). After 6 days (T_6_), an increase in microbial concentration was observed for all the treatments done, except for sodium lactate/sodium diacetate. However, the total bacterial load of meat treated with sodium lactate/sodium diacetate was not statistically different (Table S1, Supplementary Materials) from that observed in meat with 1.6% and 2.4% of tomato byproduct extract. After 9 days, all the extract concentrations, with the exception of 0.8%, had an effect statistically comparable with sodium lactate/sodium diacetate, while meat not containing extract or sodium lactate showed a further bacterial load increase.

The efficacy of fermented melon and carrot byproduct extracts on food preservation was tested on a ready-to-eat sliced vegetal product mainly composed of melon. As reported in Table 2, it is clear that, just after the preparation, there was a positive antimicrobial effect of the fermented byproduct extracts. A difference in microbial load was clear between the ready-to-eat sliced vegetal product not containing extracts and that containing byproduct fermented extracts. Indeed, in the control sample (untouched ready-to-eat sliced vegetal product), an increase in the total microbial load was observed, especially after 15 days of storage. On the contrary, the employment of fermented melon and carrot byproduct extracts at 4 log_10_ CFU/mL reduced the microbial load just after the preparation of the product, maintaining the microbial concentration throughout the storage period.

### 3.3. Effect of Fermented Tomato Byproduct Extract on Foodborne Pathogens in Minced Meat

**Figure 2:**
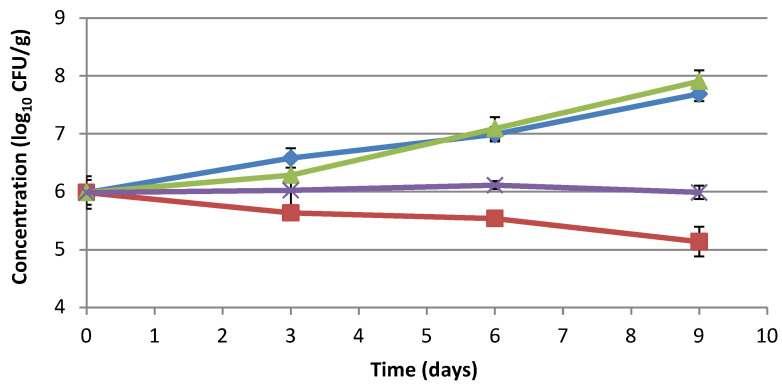
Effect of fermented tomato byproduct extract on *Salmonella* spp. in minced pork meat. Behavior of *Salmonella* spp. in minced pork meat with or without fermented tomato byproduct extract/preservatives determined through microbial challenge test. *Salmonella* spp. concentration in minced pork meat (blue line), minced pork meat with 1.6% fermented tomato byproduct extract (red line), minced pork meat with 1.2% sodium lactate (green line), and minced pork meat with 2.5% sodium lactate/sodium diacetate (96:4) (purple line). Concentration is expressed as log_1__0_ CFU/g.

Minced pork meat was also used as a food model to simulate, through microbiological challenge test, the behavior of different foodborne pathogens (*Salmonella* spp., *L. monocytogenes*, and *B. cereus*) in case of an eventual contamination. *Salmonella* spp. concentration increased in control samples over time until reaching the highest concentration after 9 days of refrigerated storage. The addition of sodium lactate did not affect *Salmonella* growth, while the addition of sodium lactate/sodium diacetate and fermented tomato byproduct extract slowed it down. The inhibitory effect of the extract was already noticeable after 3 days, whereas, after 9 days, a decrease of almost 1 log_10_ CFU/g was observed compared to T_0_ (Figure 2). After 9 days, the difference between the control sample and meat with extract was 2.55 log_10_ CFU/g. Furthermore, a significant difference in activity could be observed between fermented tomato byproduct extract and sodium lactate/sodium diacetate at the end of storage (Table S2, Supplementary Materials).

**Figure 3:**
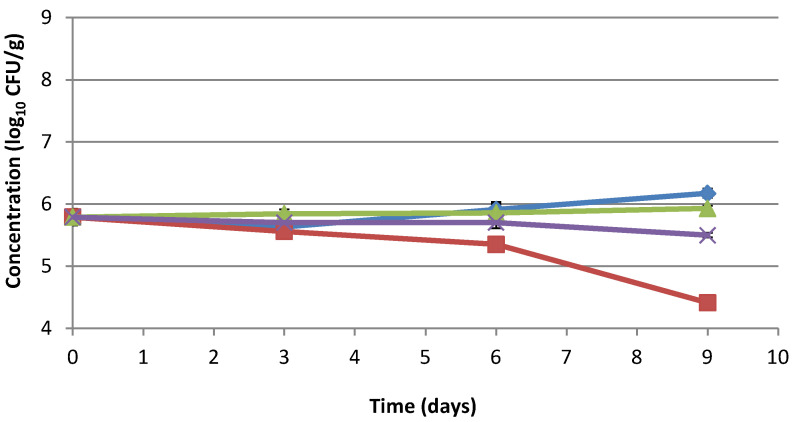
Effect of fermented tomato byproduct extract on *L. monocytogenes* in minced pork meat. Behavior of *L. monocytogenes* in minced pork meat with or without fermented tomato byproduct extract/preservatives determined through microbial challenge test. *L. monocytogenes* concentration in minced pork meat (blue line), minced pork meat with 1.6% fermented tomato byproduct extract (red line), minced pork meat with 1.2%sodium lactate (green line), and minced pork meat with 2.5% sodium lactate/sodium diacetate (96:4) (purple line). Concentration is expressed as log_10_ CFU/g.

A similar trend was followed by *L. monocytogenes*. The extract showed an inhibitory effect on *L. monocytogenes* after 9 days with a decrease in its concentration of 1.38 log_10_ CFU/g compared to T_0_. Sodium lactate/sodium diacetate was less effective against this foodborne pathogen species, while sodium lactate showed no significant effect (Figure 3, Table S3, Supplementary Materials). Overall, at the end of storage, the difference between the control sample and meat with the extract was 1.76 log_10_ CFU/g.

**Figure 4:**
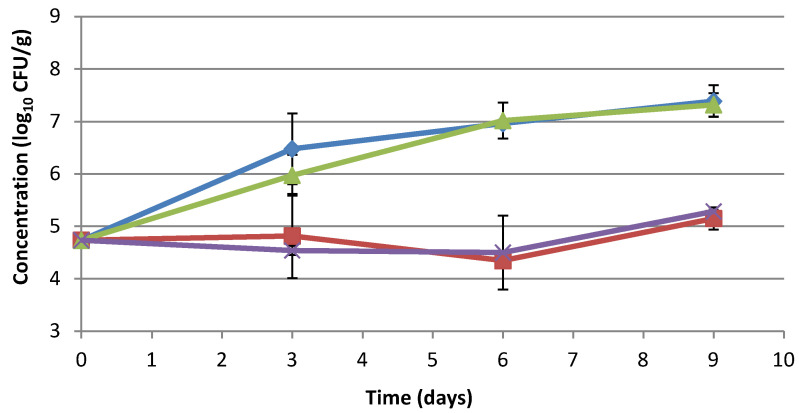
Effect of fermented tomato byproduct extract on *B. cereus* in minced pork meat. Behavior of *B. cereus* in minced pork meat with or without fermented byproduct tomato extract/preservatives determined through microbial challenge test. *B. cereus* concentration in minced pork meat (blue line), minced pork meat with 1.6% fermented byproduct tomato extract (red line), minced pork meat with 1.2% sodium lactate (green line), and minced pork meat with 2.5% sodium lactate/sodium diacetate (96:4) (purple line). Concentration is expressed as log_10_ CFU/g.

*B. cereus* was able to grow in refrigerated conditions in minced pork meat. Sodium lactate did not stop its development at the tested concentration, while sodium lactate/sodium diacetate and extract inhibited the growth, maintaining the cell number until 6 days and allowing a slight growth between days 6 and 9. At the end of storage, the difference between the control sample and minced pork meat with extract was 2.24 log_10_ CFU/g (Figure 4, Table S4, Supplementary Materials).

## 4. Discussion

Consumers are increasingly concerned about synthetic preservatives used in foodstuff [6,7], perceived as not natural. As a consequence, the demand for minimally processed foods and “cleaner” labels is rising, and the research into new and efficient natural antimicrobials is growing in importance [5,23]. During lactic acid fermentation, LAB are able to produce different compounds exerting antimicrobial activity [15,16,17] such as hydrogen peroxide, CO_2_, peptides or proteins, bacteriocins, ethanol, acids such as acid, benzoic, sorbic, and citric lactic, and diacetyl, and their antimicrobial activity may be exerted via the combined action of different metabolites on undesirable bacteria [24]. Furthermore, some phenolic compounds, well documented in the literature for their antimicrobial activity, are naturally present in raw substrates, but their content can be implemented during lactic acid fermentation [25]. LAB are also able to produce phenyllactic acids [26], compounds with a widely documented activity on pathogenic microorganisms [27,28].

Considering this background and our previous work [15], in which the extracts obtained from fermented tomato, melon, and carrot byproducts exhibited antimicrobial activity, in the present work, we wanted to further verify the efficacy of the extracts directly in food. Initially, the MBC of the same extracts was determined considering 16 strains belonging to *Salmonella* spp., *L. monocytogenes, E. coli, S. aureus, B. cereus*, and *Pseudomonas* spp. Using the susceptibility test, we identified the concentration at which each extract caused the death of microorganisms tested. Fermented tomato, melon, and carrot byproduct extracts exhibited bactericidal activity against all tested strains. Overall, the highest efficacy (ranging from 25 to 3.13 mg/mL) was observed at the lowest bacterial concentration tested (2 log_10_ CFU/mL). However, for most strains, the same extract concentration also resulted bactericidal at higher bacterial concentrations (4, 6, and 8 log_10_ CFU/mL). Different studies reported in the literature focused their attention on natural antimicrobial activity, often based on essential oils. However, fewer studies took into consideration plant extracts [29,30,31,32,33], with even fewer considering the antimicrobial activity of fermented extracts [34,35,36] and only one exploiting the fermentation of byproducts to produce extracts [37], outside of our previous work [15]. These studies reported the effective antimicrobial activities of fermented extracts. Di Onofrio et al. (2019) [34] reported the effect of spontaneous fermented garlic extract on two strains of *Pseudomonas aeruginosa*. They reported an MIC of 4%, a value that was higher than the MBC of fermented tomato and carrot extracts observed in the present study for strains of the same genus (MBC ranged from 25 to 6.25 mg/mL). Pine needles were spontaneously fermented by Yim et al. (2006), showing activity against Gram-positive (*S. aureus* and *B. cereus*) and Gram-negative (*Salmonella* Typhimurium and *P. aeruginosa*) bacteria [35]. *Codonopsis lanceolata* was fermented with LAB by He et al. (2011) [36], and, after fermentation, a huge reduction in minimum inhibitory concentration was observed compared to the nonfermented plant, confirming that lactic acid fermentation positively affects the antimicrobial activity of different raw plant materials. Moreover, ginger marc was fermented with *Lactiplantibacillus plantarum* by Eom et al. (2017) [37], and the fermented product showed antimicrobial activity against *S. aureus* and *E. coli.* Considering a potential application to extend the shelf-life of foods, the efficacy of fermented tomato byproduct extract was tested in minced pork meat at different concentrations (0.8%, 1.2%, 1.6%, and 2.4%). We observed that the extract maintained a total microbial load lower than the control (minced pork meat without extract), and its activity was comparable (at 1.6% and 2.4%) with that of sodium lactate/sodium diacetate, a common preservative used for meat preservation [38,39]. Instead, the activity of fermented melon and carrot byproduct extracts (1.2%) was tested on a ready-to-eat sliced vegetal product throughout its shelf life. In this case, we also observed that extracts maintained a markedly lower total microbial load than the control, for all 30 days monitored. After the evaluation of the positive effect against spoilage microorganisms, we wanted to test in situ the effect against foodborne pathogens. In particular, we determined the efficacy of fermented tomato byproduct extract at 1.6% in minced pork meat on *Salmonella*, *L. monocytogenes*, and *B. cereus*, during refrigerated storage. Results of the microbial challenge test highlighted that *Salmonella* and *L. monocytogenes* were not able to grow when the extract was present, decreasing over 9 days, while *B. cereus* was inhibited until 6 days before a slight increase was observed between days 6 and 9 of storage. To the best of our knowledge, no study has reported the activity in pork meat of extracts obtained after lactic acid fermentation. However, different plant extracts were recently tested for pork meat preservation. Swamp cranberry fruit and pomace extracts were used at 2.5% in minced pork meat, showing a decrease in *L. monocytogenes* and *Salmonella enteritidis* of 4 log_10_ CFU/g after 4 days of storage [40]. Another study [41] tested oakwood extract as a preservative at different concentrations (0.05%, 0.5%, and 1%) in pork patties, which did not have an effect on the total microbial count, unlike the fermented tomato, melon, and carrot byproduct extracts in this study, which exerted an inhibitory effect. In 2016, Novak and colleagues tested the antimicrobial activity of cherry and blackcurrant leaf extracts (0.5% and 1%, respectively) in vacuum-packed pork sausages in refrigerated storage conditions [42]. Mesophilic bacteria, psychotropic bacteria, LAB, and Enterobacteriaceae were not affected by the presence of the two extracts, whereas, after only 28 days of storage, *Pseudomonas* growth was lower compared to the control without extracts.

## 5. Conclusions

From the different reports available in literature, it is recognized that plants, as well as microorganisms, can be sources of antimicrobial compounds which can be applied for food preservation. However, the employment of both, exploiting the lactic acid fermentation process, is practically unexplored, with very few studies considering this opportunity [15,34,35,36]. Despite the low number of papers on this topic, evidence suggests an improvement of antimicrobial activity during fermentation. To the best of our knowledge, no one, except for Ricci et al. (2019) [15], has used tomato, melon, and carrot byproducts as raw matrices fermentable for the recovery of antimicrobial compounds. Therefore, the present paper suggests a novel idea for the exploitation of these byproducts by coupling them with lacto-fermentation, recovering extracts characterized by antimicrobial activity. All these extracts demonstrated activity both “in vitro” and in foodstuff, showing that byproduct lacto-fermentation can be a strategy for the recovery of antimicrobials which can be applied for food preservation. Starting from the results achieved in this study, these extracts can be employed as natural preservatives to improve the safety and shelf-life of different categories of foodstuff, in line with the demand of consumers for cleaner and simpler labels replacing artificial preservatives perceived as “chemicals”.

## 6. Patents

Italian patent n. 102019000006815 (14 May 2019) with international extension PCT/IB2020/054520 (13 May 2020) is pending.

## Figures and Tables

**Table 1:** Minimum bactericidal concentration (MBC) of fermented tomato, melon, and carrot byproduct extracts (mg/mL) on *Salmonella* spp., *L. monocytogenes*, *E. coli*, *S. aureus*, *B. cereus*, and *Pseudomonas* spp. Different microbial concentrations were tested for each extract (8, 6, 4, and 2 log_10_ CFU/mL) (ATCC: the American Type Culture Collection; DSMZ: Deustsche Sammlung von Mikroorganismen und Zellkulturen GmbH; BCCM/LMG: the Belgian Coordinated Collection of Microorganisms; NCYC: the National Collection of Type Cultures).

	Tomato Byproduct Fermented Extract				Melon Byproduct Fermented Extract				Carrot Byproduct Fermented Extract			
Microorganism	8 log_10_	6 log_10_	4 log_10_	2 log_10_	8 log_10_	6 log_10_	4 log_10_	2 log_10_	8 log_10_	6 log_10_	4 log_10_	2 log_10_
*Salmonella* ATCC 1408	25	25	25	12.5	25	25	25	25	12.5	12.5	12.5	12.5
*Salmonella* Rissen	25	25	25	25	25	25	25	25	50	6.25	6.25	6.25
*Salmonella* suini	50	50	50	25	25	25	25	25	50	12.5	12.5	12.5
*L. monocytogenes* LM30	50	25	25	12.5	25	25	25	25	25	12.5	12.5	6.25
*L. monocytogenes* LMG 21264	100	25	25	12.5	25	25	25	12.5	>50	25	6.25	6.25
*L. monocytogenes* LMG 13305	100	25	25	12.5	25	25	25	25	50	25	12.5	6.25
*E. coli* DSM 9025	25	25	25	25	25	25	25	25	25	6.25	6.25	6.25
*E. coli* DSM 10973	25	25	25	12.5	25	25	25	25	25	6.25	6.25	6.25
*E. coli* POM 1048	25	25	25	12.5	50	25	25	25	12.5	12.5	12.5	6.25
*S. aureus* 9393	25	25	25	12.5	25	25	25	25	12.5	12.5	12.5	6.25
*S. aureus* 6538	25	25	12.5	12.5	25	25	25	25	12.5	12.5	12.5	3.13
*S. aureus* ATCC 19095	12.5	12.5	12.5	12.5	25	25	25	25	12.5	12.5	12.5	6.25
*B. cereus* 31	25	25	25	12.5	25	25	25	25	12.5	12.5	12.5	6.25
*Pseudomonas* spp. 5003	25	25	25	12.5	50	50	50	25	12.5	12.5	6.25	6.25
*Pseudomonas* spp. 5004	25	25	25	12.5	50	50	25	12.5	25	6.25	6.25	6.25
*Pseudomonas* spp. 5005	25	25	25	12.5	25	25	25	25	12.5	6.25	6.25	6.25

**Table 2:** Total microbial load ± standard deviation (log_10_ CFU/g) in ready-to-eat sliced vegetal product with (1.2%) or without fermented melon and carrot byproduct extracts after its preparation (T_0_) and after 7 (T_7_), 15 (T_15_), and 30 (T_30_) days of storage.

	T_0_	T_7_	T_15_	T_30_
Ready-to-eat sliced vegetal product	4.04 ± 0.47	4.12 ± 0.06	7.22 ± 0.07	6.48 ± 0.16
Ready-to-eat sliced vegetal product + fermented melon byproduct extract	<1	<1	<1	<1
Ready-to-eat sliced vegetal product + fermented carrot byproduct extract	<1	<1	1.83 ± 0.40	1.44 ± 0.62

## Data Availability

Not applicable.

## Supplementary Materials

The following are available online at https://www.mdpi.com/article/10.3390/foods10051092/s1: Table S1. Effect of fermented extracts on food preservation; Table S2. Effect of fermented tomato extracts on *Salmonella* spp.; Table S3. Effect of fermented tomato extracts on *L. monocytogenes*; Table S4. Effect of fermented tomato extracts on *B. cereus*.
