# Supplementary material for: Antimicrobial Activity of Fermented Vegetable Byproduct Extracts for Food Applications
Source: Foods. 2021 May 14;10(5):1092. doi: 10.3390/foods10051092 (PMC8156661; doi:10.3390/foods10051092)
Supplement: Supplementary file 1 [file foods-10-01092-s001.zip › foods-1194128-supplementary.pdf]

Supplementary Materials

Table S1. Effect of fermented extracts on food preservation. Total bacterial load (Log CFU/g) in minced pork meat added or not with fermented tomato extract/preservative. ANOVA test was used to evaluate differences among treatments (addition or not of fermented extracts/preservatives) at the same time of storage.

**Sample**	**T_0**	**T_3**	**T_6**	**T_9**
Minced meat (control)		8.51 ± 0.14^{e}	9.68 ± 0.41^{d}	10.48 ± 0.03^{c}
Fermented extract (0.8 %)		7.32 ± 0.00^{c}	8.68 ± 0.30^{c}	8.51 ± 0.51^{b}
Fermented extract (1.2 %)		6.61 ± 0.04^{b}	7.92 ± 0.03^{b}	8.05 ± 0.01^{ab}
Fermented extract (1.6 %)	6.94 ± 0.10	6.43 ± 0.03^{b}	7.59 ± 0.01^{ab}	7.93 ± 0.11^{a}
Fermented extract (2.4 %)		6.04 ± 0.03^{a}	7.35 ± 0.15^{a}	7.61 ± 0.04^{a}
1.2 % of sodium lactate		8.09 ± 0.04^{d}	9.47 ± 0.16^{d}	10.04 ± 0.07^{c}
2.5 % of sodium lactate/sodium diacetate		7.31 ± 0.19^{c}	7.23 ± 0.08^{a}	7.63 ± 0.49^{a}

Table S2. Effect of fermented tomato extracts on *Salmonella* spp. *Salmonella* spp. concentration (Log CFU/g) in minced pork meat added or not with fermented tomato extract/preservative. ANOVA test was used to evaluate differences among treatments (addition or not of fermented extracts/preservatives) at the same time of storage.

**Sample**	**T_0**	**T_3**	**T_6**	**T_9**
Minced meat (control)		6.58 ± 0.17^{c}	6.90 ± 0.12^{c}	7.69 ± 0.12^{c}
Fermented extract (1.6 %)		5.64 ± 0.08^{a}	5.54 ± 0.07^{a}	5.14 ± 0.25^{a}
1.2 % of sodium lactate	5.99 ± 0.05	6.29 ± 0.29^{bc}	7.09 ± 0.20^{c}	7.91 ± 0.19^{c}
2.5 % of sodium lactate/sodium diacetate		6.02 ± 0.26^{ab}	6.11 ± 0.07^{b}	5.99 ± 0.12^{b}

Table S3. Effect of fermented tomato extracts on *L. monocytogenes*. Behaviour of *L. monocytogenes* (Log CFU/g) in minced pork meat added or not with fermented tomato extract/preservative. ANOVA test was used to evaluate differences among treatments (addition or not of fermented extracts/preservatives) at the same time of storage.

**Sample**	**T_0**	**T_3**	**T_6**	**T_9**
Minced meat (control)		5.64 ± 0.01^{a}	5.91 ± 0.08^{c}	6.18 ± 0.07^{d}
Fermented extract (1.6 %)		5.56 ± 0.04^{a}	5.35 ± 0.02^{a}	4.41 ± 0.02^{a}
1.2 % of sodium lactate	5.79 ± 0.13	5.84 ± 0.07^{b}	5.86 ± 0.18^{bc}	5.93 ± 0.04^{c}
2.5 % of sodium lactate/sodium diacetate		5.70 ± 0.16^{ab}	5.70 ± 0.09^{b}	5.50 ± 0.05^{b}

Table S4. Effect of fermented tomato extracts on *B. cereus*. Behaviour of *B. cereus* (Log CFU/g) in minced pork meat added or not with fermented tomato extract/preservative. ANOVA test was used to evaluate differences among treatments (addition or not of fermented extracts/preservatives) at the same time of storage.

**Sample**	**T_0**	**T_3**	**T_6**	**T_9**
Minced meat (control)		6.48 ± 0.67^{b}	6.96 ± 0.06^{b}	7.39 ± 0.30^{b}
Fermented extract (1.6 %)	4.74 ± 0.06	4.82 ± 0.80^{a}	4.35 ± 0.07^{a}	5.15 ± 0.21^{a}
1.2 % of sodium lactate		5.97 ± 0.39^{b}	7.02 ± 0.34^{b}	7.32 ± 0,22^{b}

2.5 % of sodium lactate/sodium diacetate	4.54 ± 0.09[a]	4.50 ± 0.71[a]	5.28 ± 0.02[a]
